# Reasons for Nonadherence to the Direct Oral Anticoagulant Apixaban: A Cross-Sectional Survey of Atrial Fibrillation Patients

**DOI:** 10.1016/j.jacadv.2022.100175

**Published:** 2023-01-27

**Authors:** Derjung M. Tarn, Kevin Shih, Chi-hong Tseng, Alveena Thomas, Janice B. Schwartz

**Affiliations:** a Department of Family Medicine, David Geffen School of Medicine at UCLA, University of California-Los Angeles, Los Angeles, California, USA; b Division of General Internal Medicine/Health Services Research, Department of Medicine, David Geffen School of Medicine at UCLA, University of California-Los Angeles, Los Angeles, California, USA; c Division of Geriatrics, Department of Medicine, University of California-San Francisco, San Francisco, California, USA; d Division of Clinical Pharmacology, Departments of Medicine and Bioengineering and Therapeutic Sciences, University of California-San Francisco, San Francisco, California, USA

**Keywords:** anticoagulation, apixaban, atrial fibrillation, direct-acting oral anticoagulant, medication adherence, stroke prevention

## Abstract

**Background:**

Nonadherence to direct oral anticoagulants to prevent stroke occurs in up to 40% of atrial fibrillation patients. Underlying reasons are poorly understood.

**Objectives:**

This study quantified patient-reported reasons for nonadherence and identified strategies to improve adherence.

**Methods:**

This is a cross-sectional survey of atrial fibrillation patients in 2 academic health systems who reported apixaban nonadherence. We examined patient-reported reasons for nonadherence and level of nonadherence (assessed by a validated 3-item adherence measure) using a multivariable logistic regression model.

**Results:**

Of 419 study patients, 41.5% were women. The mean age was 71.1 ± 10 years and mean CHA_2_DS_2_VASc score was 3.2 ± 1.6. About two-thirds had adherence scores ≥80 (mild nonadherence) and one-third scores <80 (poor adherence). In all groups, forgetfulness contributed to nonadherence. Attitudes/beliefs associated with adherence score <80 included: not believing apixaban was needed (odds ratio [OR]: 12.24 [95% CI: 2.25-66.47]); medication cost (OR: 3.97 [95% CI: 1.67-9.42]); and fear of severe bleeding (OR: 3.28 [95% CI: 1.20-8.96]). Strategies that patients with adherence scores <80 selected as helping “a great deal/a lot” to increase adherence included bloodwork to evaluate efficacy (56%), physician counseling about adherence (55%), and having a reversal agent (39%). Almost one-half of all patients did not disclose nonadherence to their providers.

**Conclusions:**

Patients may not disclose their nonadherence to prescribers, and attitudes related to apixaban nonadherence differ among patients with mild nonadherence versus poor adherence. While all patients may benefit from strategies to address forgetfulness, concerns related to the purpose of apixaban, cost, and bleeding risk may require special attention in those with poor adherence.

Warfarin was the anticoagulant initially recommended to prevent strokes in patients with atrial fibrillation (AF).^1^ Nonadherence to warfarin and challenges related to maintaining anticoagulation within target international normalized ratio ranges, however, have limited its clinical utility.^2^ The introduction of direct-acting oral anticoagulants (DOACs) brought hope for improved adherence in patients with nonvalvular AF, with the lack of requisite monitoring and simplified dosing resulting from reduced food and drug interactions.^2^^,^^3^ Currently, DOACs are the first-line guideline-recommended medications for stroke prevention in patients with nonvalvular AF.^4^, ^5^, ^6^, ^7^, ^8^ Yet real-world evidence suggests that adherence to DOACs remains poor in up to about 40% of patients prescribed DOACs.^9^, ^10^, ^11^, ^12^, ^13^

Information on the reasons underlying nonadherence to DOACs is lacking. Studies using administrative databases and mathematical modeling have identified patient characteristics, therapy-related, condition-related, socioeconomic, and health system factors associated with poor adherence to oral anticoagulants,^9^^,^^14^, ^15^, ^16^, ^17^, ^18^, ^19^, ^20^, ^21^ but cannot provide an understanding of why patients miss doses or elucidate underlying patient perceptions, beliefs, and knowledge that may contribute to nonadherence. Reasons for nonadherence to warfarin likely differ from those for DOAC nonadherence. Warfarin is covered by Medicare and most insurance plans, while coverage for DOACs can be variable. A major concern regarding warfarin is the inconvenience of regular monitoring.^22^ Many warfarin patients receive follow-up in specialized anticoagulation clinics with patient education programs that are not part of the routine care of patients with AF treated with DOACs. An understanding of why patients are nonadherent to DOACs is crucial for interventions targeting DOAC adherence in the absence of specialized monitoring and education programs.

We previously conducted semistructured interviews with patients with nonvalvular AF and identified major reasons they chose to skip doses or to discontinue the DOAC apixaban (the most frequently prescribed DOAC for nonvalvular AF).^23^ These interviews revealed reasons for intentional nonadherence that included medication cost, fear of and experience with bleeding, lack of AF symptoms, beliefs that skipping doses is safe, and confusion about a lack of measurable effects.^23^ The semistructured interviews elucidated the range of reasons people raise for nonadherence to DOACs, but did not yield quantitative information regarding how often people cite these reasons for nonadherence. In addition, the prior interviews did not assess potential patient-identified strategies for improving adherence. The objectives of this study were to quantify: 1) reasons for nonadherence to apixaban in patients with AF; and 2) patient preferences for potential strategies to promote adherence.

## Methods

### Design/setting

This was a cross-sectional survey of patients with AF who reported nonadherence to apixaban. Surveys were administered from June to November 2021 at 2 academic medical centers, the University of California-Los Angeles (UCLA) and the University of California-San Francisco (UCSF). The UCLA Institutional Review Board approved the study protocol, with the UCSF relying on the UCLA Institutional Review Board.

### Participants

Potential study participants receiving apixaban between August 2019 and August 2021 were identified via large-scale electronic health record (EHR) data extractions at each site and screened for eligibility. Those with email addresses were emailed an electronic Qualtrics survey link, while those without email addresses were mailed paper copies of the survey with a postage-paid return envelope. Nonrespondents received up to 3 to 5 reminders via emails/postal mailings and/or 1 telephone call. Respondents received a $25 gift card for survey completion.

Screening questions for study eligibility were completed by all respondents. Eligible patients were adults prescribed apixaban for AF or atrial flutter who reported “not taking Eliquis” or “sometimes” or “often” missing apixaban doses without a physician’s recommendation. Only eligible patients were directed to the survey.

### Survey contents (SUPPLEMENTAL Appendix)

#### Reasons for nonadherence to apixaban

Pre-survey, we performed cognitive interviews with 10 patients to test the comprehensibility, mutual exclusiveness, and adequate representation of questions and response options regarding reasons for patient nonadherence. Questions were iteratively refined based on patient feedback. On the final survey, questions regarding the reasons for patient nonadherence asked patients to select both the most important reason and all applicable responses from a list of 10 potential reasons for nonadherence to apixaban. Patients were also given an option to check ‘other’ and write-in reason(s).

Cost-related nonadherence was assessed using questions modified from an existing validated 3-item measure. Patients were asked whether they “skipped apixaban (Eliquis) doses,” “have taken less apixaban (Eliquis),” or “delayed filling apixaban (Eliquis)” to save money during the last 12 months.^24^^,^^25^ Any “yes” response indicated cost-related nonadherence.

#### Patient perceptions

Questions on patient perceptions about goals for apixaban use and about stroke and bleeding risk were adapted from existing studies and from our previous interviews with patients.^9^^,^^23^^,^^26^^,^^27^ These questions asked about the level of risk of having a stroke in the next year that would motivate patients to take apixaban, and about whether they had greater fear of having a stroke or bleeding. Patients also were asked about their agreement or disagreement with the statement: “Eliquis prevents strokes in people with atrial fibrillation” on a 5-point Likert scale.

#### Preferences for strategies to promote adherence

Five items asked patients to rate different strategies to promote adherence on helpfulness for taking apixaban “exactly the way [their] doctor advised.” Response options were based on 5-point Likert scales. We also queried patients about whether they told their physician about their nonadherence (yes/no).

#### Patient characteristics

Other survey items queried patient characteristics such as basic demographics and health literacy.^28^^,^^29^ Patients also reported on type of AF (paroxysmal; chronic, persistent, or permanent; or unknown), current number of prescription medications taken, health conditions needed to calculate their CHA_2_DS_2_-VASc score (estimation of stroke risk for AF patients), and prior use of warfarin.

#### Outcome measurement: adherence to apixaban

Self-reported adherence to apixaban was assessed with an existing and validated 3-item measure.^30^ This measure performed well in validation against electronic drug monitoring and demonstrated excellent internal consistency in field testing and validation studies (Cronbach’s α = 0.83-0.87).^30^^,^^31^ Patients reported on the number of days they missed at least one dose of apixaban (0-30 days), “how often” and “how good a job” they did with taking apixaban in the way they were supposed to in the last 30 days (6-point Likert scales). The first item was coded into “days taken,” after which each item was linearly transformed to a 0 to 100-point scale with 100 representing the best adherence. The mean of the 3 scores was taken to get an equally weighted aggregate score.

### Analyses

Descriptive statistics were calculated to describe patient characteristics, reasons for nonadherence, patient perceptions, and strategies to promote adherence. Bivariate analyses assessed relationships of these variables with adherence to apixaban using analysis of variance and t-tests, as appropriate for categorical and continuous variables. The adherence score was dichotomized into <80 (poor adherence) and ≥80 (mild nonadherence) based on cutoffs commonly used in the literature.^32^ Those with adherence scores <80 were separated into those with scores <60 and scores from 60 to 79 to assess whether those with the poorest adherence differed from those with higher adherence scores.

We modeled reasons for nonadherence to apixaban on adherence using multivariable logistic regression, adjusting for patient demographics and health characteristics. Continuous variables included: age, number of prescription medications, health literacy, and CHA_2_DS_2_-VASc score. Categorical variables included: patient sex, race/ethnicity, education, prior warfarin use, cost-related nonadherence, and reasons for nonadherence to apixaban. Adherence was modeled as a dichotomous outcome (adherence scores <80 and ≥80). All analyses were performed using R version 4.1.2.

## Results

Of 8,365 survey invitations sent, 2,439 responded, 2066 reported having AF/flutter and receiving a prescription for apixaban, of which 428 (20.7%) reported nonadherence, giving an eligibility rate of 17.5% (428/2,439). Of 428 eligible patients, 419 (97.8%) completed the survey. Applying the eligibility rate to those for whom we were unable to determine eligibility (n = 8,365-2,439 = 5,926), the net response rate was 28.6% [419/(428 + [0.175 × 5,926]).

Overall, patients had a mean age of 71.1 years (SD = 10.0), more than half were male, 83.5% were White, and two-thirds had at least a college degree (Table 1). Almost half reported that their symptoms were paroxysmal. Two-thirds of the patients had adherence scores ≥80 (n = 278), 20% had adherence scores of 60 to 79 (n = 84), and 14% had scores <60 (n = 57). CHA_2_DS_2_VASc scores did not differ significantly among groups. Those with adherence scores ≥80 reported taking more prescription medications than the other groups (median of 5, 4, and 4 medications for adherence scores ≥80, 60 to 79, and <60, respectively; *P* = 0.009).

**Table 1:** Patient Characteristics: Overall and by Adherence Score

	All Patients (N = 419, 100%)	Adherence Score <60 (n = 57, 13.6%)	Adherence Score 60-79 (n = 84, 20.1%)	Adherence Score ≥80 (n = 278, 66.3%)	*P* Value for Comparison Among the 3 Adherence Groups
Age, y	71.1 ± 10.9 (25-99)	68.6 ± 10.6 (37-91)	71.2 ± 12.3 (36-96)	71.6 ± 10.4 (25-99)	0.20
Female	174 (41.5)	19 (33.3)	34 (40.5)	121 (43.5)	0.35
Race/ethnicity					
Asian	36 (8.6)	6 (10.5)	7 (8.3)	23 (8.3)	0.80
Black	19 (4.5)	2 (3.5)	6 (7.1)	11 (4)	0.43
Hispanic	29 (6.9)	5 (8.8)	7 (8.3)	17 (6.1)	0.61
White	350 (83.5)	46 (80.7)	70 (83.3)	234 (84.2)	0.79
Other	14 (3.3)	3 (5.3)	1 (1.2)	10 (3.6)	0.43
Education					0.97
High school or less	31 (7.4)	4 (7.0)	6 (7.1)	21 (7.6)	
Some college	109 (26)	15 (26.3)	18 (21.4)	76 (27.3)	
College graduate	105 (25.1)	15 (26.3)	22 (26.2)	68 (24.5)	
Graduate school	174 (41.5)	23 (40.4)	38 (45.2)	113 (40.6)	
Number of prescription medications	5 (3-7)	4 (2-6)	4 (3-6)	5 (4-7)	0.009
Health literacy^a^	3.4 ± 0.9	3.4 ± 0.8	3.2 ± 1.0	3.5 ± 0.8	0.03
Prior warfarin use	97 (23.2)	9 (15.8)	18 (21.4)	70 (25.2)	0.31
CHA_2_DS_2_VASc score	3.2 ± 1.6	2.7 ± 1.5	3.3 ± 1.6	3.2 ± 1.5	0.09
Prior history of stroke or transient ischemic attack	61 (14.6)	5 (8.8)	14 (16.7)	42 (15.1)	0.74
Atrial fibrillation type					0.03
Paroxysmal	192 (45.8)	23 (40.4)	42 (50)	127 (45.7)	
Persistent/permanent	92 (22)	11 (19.3)	12 (14.3)	69 (24.8)	
Unknown	135 (32.2)	23 (40.4)	30 (35.7)	82 (29.5)	
Emergency department or urgent care visit in the past 12 months for bleeding	16 (4.0)	4 (7.0)	1 (1.3)	11 (4.2)	0.23

Values are mean ± SD (range), n (%), median (IQR), or mean ± SD.

a Health literacy ranges from 0 to 4 with higher numbers indicating greater health literacy.^28^^,^^29^

### Reasons for nonadherence

Overall, patients selected reasons for nonadherence such as forgetfulness (80%), not always experiencing AF symptoms (13%), fear of severe bleeding (9%), cost (8%), bothersome bruising (7%), not believing that apixaban was needed (4%), and believing that it was acceptable to occasionally skip doses (4%) (Central Illustration).

**Central Illustration:**
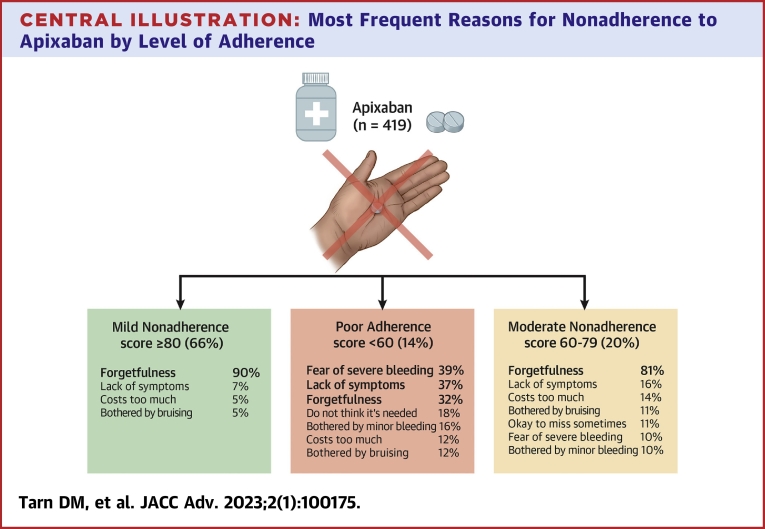
**Most Frequent Reasons for Nonadherence to Apixaban by Levelof Adherence** The reasons for nonadherence are not mutually exclusive.

**Figure 1:**
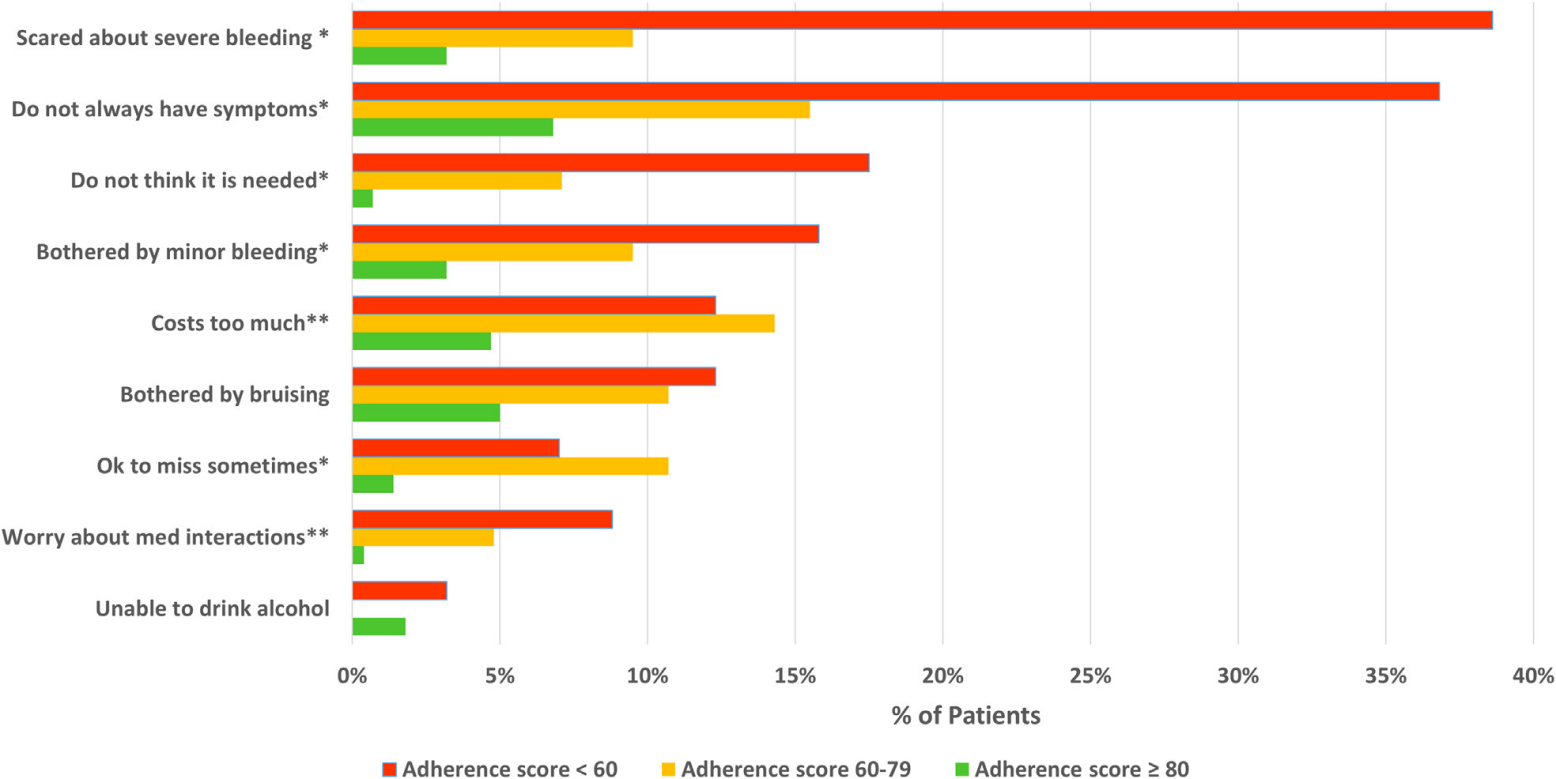
**Patient Self-Reported Reasons for Nonadherence to Apixaban (Excluding Forgetfulness), by Adherence Score** Patients selected all applicable reasons from a list. N = 419 (n = 57, n = 84, and n = 278 for those with adherence scores <60, 60-79, and ≥80, respectively). ∗*P* < 0.001 for differences among the three groups, ∗∗*P* < 0.01 for differences among the three groups

Among patients with *mild nonadherence* (adherence scores ≥80), 90% reported forgetfulness. For this group, as shown in Figure 1, attitudes and beliefs contributed less frequently to nonadherence than for those with poor adherence. While not always having symptoms was cited by 7% and bruising by 5% of those with mild nonadherence, all other reasons were selected by <5% of patients.

For patients with *poor adherence* (adherence scores <80), attitudes and beliefs contributed much more frequently to nonadherence. This group was less likely than those with adherence scores ≥80 to cite forgetfulness as a cause of their nonadherence (81% of those with adherence scores 60 to 79 and 32% of those with scores <60 cited forgetfulness). Those reporting the poorest adherence (adherence scores <60) worried most about severe bleeding (39%), noted that they did not always have symptoms (37%), did not think apixaban was needed (18%), and had issues related to cost (12%) and bruising (12%). Those with adherence scores from 60 to 79 experienced the most difficulty with cost (14%), most often believed it was acceptable to occasionally miss taking apixaban (11%), and none had concerns about alcohol consumption while taking apixaban (0%). Those with adherence scores <60 selected slightly more reasons for nonadherence, mean of 1.9 ± 1.1, compared to 1.7 ± 1.1 and 1.2 ± 0.6 reasons for those with adherence scores of 60 to 79 and ≥80, respectively (*P* < 0.001).

**Figure 2:**
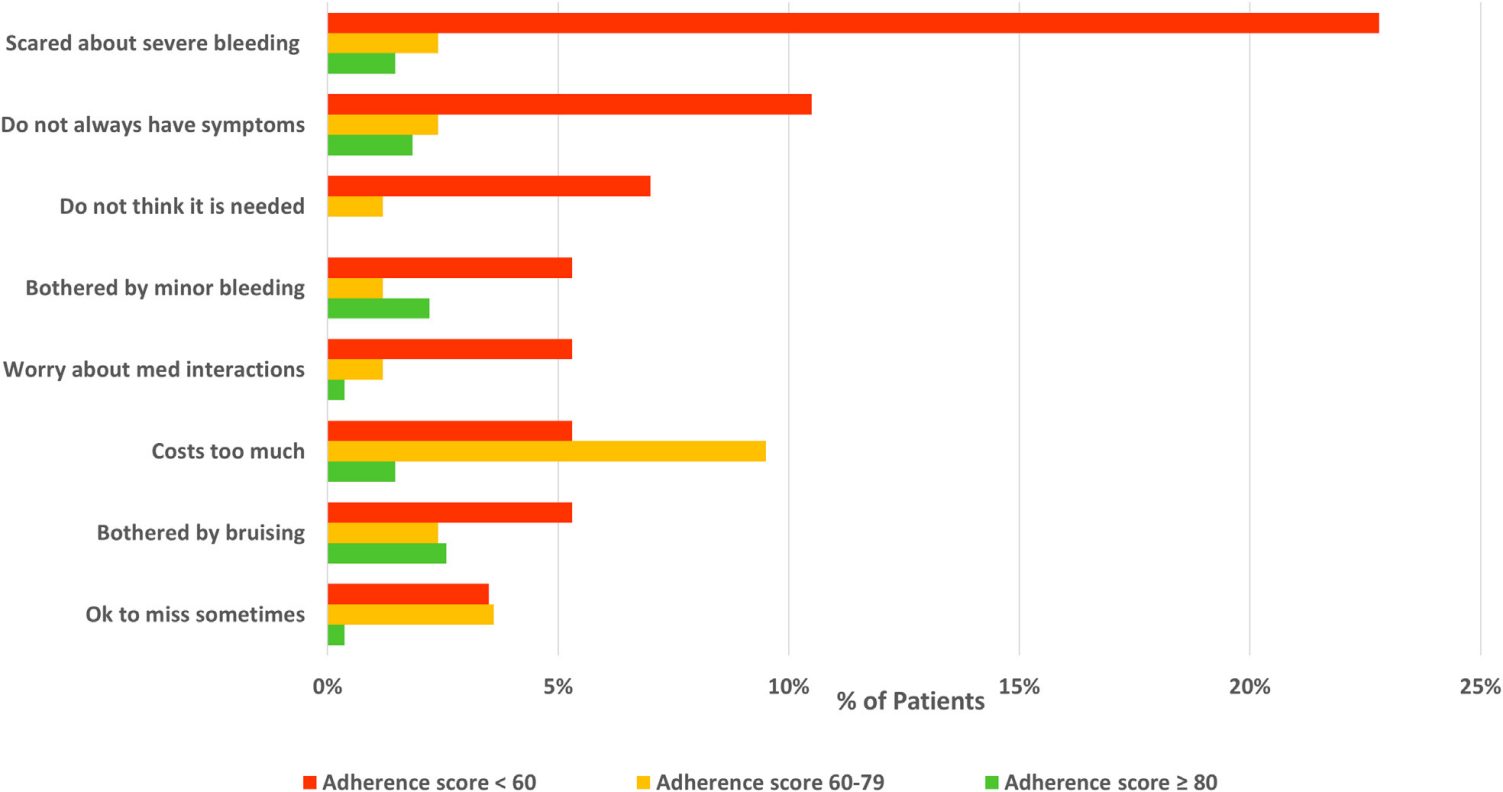
**Single Most Important Reason for Nonadherence to Apixaban (Excluding Forgetfulness), by Adherence Score** n = 57, n = 84, and n = 278 for adherence scores <60, 60 to 79, and ≥80, respectively; *P* < 0.001 for all differences among the 3 groups.

The single most important reason selected by patients for their nonadherence was forgetfulness (74.8% of all patients; 84%, 70%, and 32% of patients with adherence scores ≥80, 60 to 79, and <60, respectively). Reasons related to patient attitudes and beliefs are shown in Figure 2. Patients with the poorest adherence more frequently selected worries about severe bleeding, lack of symptoms, the need for apixaban, worries about minor bruising, and medication interactions than the other groups.

#### Multivariable analyses

Multivariable logistic regression analyses demonstrated that reasons associated with adherence scores <80 included: patients believing they did not need to take apixaban (OR: 12.24 [95% CI: 2.25-66.47), cost-related nonadherence (OR: 3.97 [95% CI: 1.67-9.42]), fear of a severe bleed (OR: 3.28 [95% CI: 1.20-8.96]), and lower health literacy (OR: 0.67 [95% CI: 0.49-0.92]) (Table 2). Other variables included in the model (age, number of prescription medications, health literacy, CHA_2_DS_2_-VASc score, sex, race/ethnicity, education, prior warfarin use, and other reasons for nonadherence to apixaban [see Methods and Figure 1]) were not statistically significant.

**Table 2:** Multivariable Logistic Model Predicting Adherence Scores <80, Including Patient Demographic and Health Characteristics, and Reasons for Nonadherence

Age	0.98 (0.95–1.01)
Male	1.24 (0.73–2.10)
Female	Reference group
Race	
Asian	1.55 (0.66–3.62)
Black	1.59 (0.47–5.31)
Hispanic	1.54 (0.60–3.99)
White	Reference group
Other	0.57 (0.12–2.58)
Education	
Graduate school	0.74 (0.40–1.37)
College graduate	Reference group
High school or less	0.49 (0.15–1.63)
Some college	0.54 (0.27–1.10)
# prescription medications	0.91 (0.83–1.00)
Prior history of warfarin use	0.62 (0.31–1.21)
CHA_2_DS_2_VASc score	1.15 (0.94–1.40)
Reasons for nonadherence	
I am not supposed to drink alcohol when I take Eliquis	0.11 (0.00–3.16)
I have had bruising from Eliquis that bothers me	1.15 (0.39–3.36)
I do not always have symptoms of atrial fibrillation	1.19 (0.51–2.80)
I do not think I need to take Eliquis	**12.24(2.25–66.47)**
It is okay to miss taking Eliquis here or there because it stays in my body	2.84 (0.78–10.36)
I am scared that Eliquis might cause bleeding that is severe or that I cannot control	**3.28(1.20–8.96)**
I forget to take Eliquis	0.44 (0.19–1.01)
I worry that Eliquis will cause problems with my other medicine	10.24 (0.98–106.59)
I have had minor bleeding from Eliquis that bothers me	2.23 (0.71–7.02)
Other	1.96 (0.54–7.04)
Cost-related nonadherence	**3.97(1.67–9.42)**
Health literacy	**0.67(0.49–0.92)**

Values are OR (95% CI). **Bold** indicates reasons found to be significant by multivariable logistic regression.

### Patient perceptions

Almost 80% of patients feared having a stroke more than having severe bleeding, while 7% feared bleeding more and 14% were unable to decide. Of those with the poorest adherence (adherence scores <60), only two-thirds feared stroke more than bleeding. In this group, 16% feared bleeding more, compared to 4% and 6% of patients with adherence scores of 60 to 79 and ≥80, respectively (*P* = 0.04). Patients with the poorest adherence also were less likely to agree that apixaban prevents strokes (61%, versus 69% and 79% of those with adherence scores 60-79 and ≥80, respectively; *P* = 0.002).

When asked about the amount of risk of stroke that would warrant taking apixaban, 30% (n = 120) did not know and 30% (n = 122) indicated that their risk of stroke would not change their apixaban use (n = 122). Of risk levels presented, 14% (n = 57) chose >2% risk, 6% (n = 23) chose each of 5% and 10%, 4% (n = 15) chose 25%, and 10% (n = 42) chose 50%. There were no differences in responses based on adherence.

### Strategies to promote apixaban adherence

**Figure 3:**
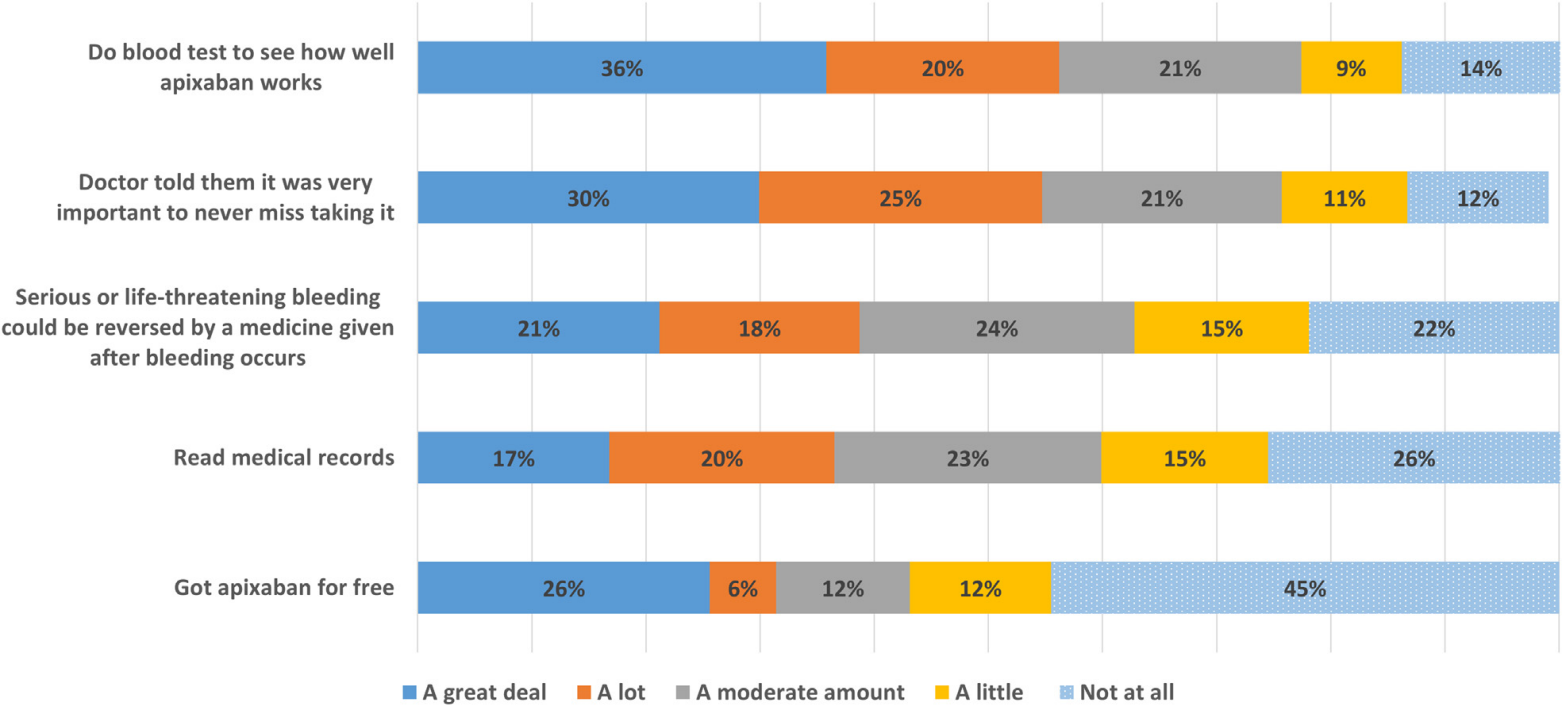
**Evaluations of Strategies to Increase Adherence Among PatientsWith Adherence Scores <80 (N = 137)**

We evaluated responses regarding potential strategies to promote adherence in patients with adherence scores <80. As shown in Figure 3, more than half indicated that strategies they believed would help a great deal/a lot to enhance their adherence included doing bloodwork to assess medication efficacy and having their doctor tell them that it was very important not to miss taking any dose of their apixaban. About one-third endorsed the ability to read their medical records, getting apixaban for free, and having the availability of a reversal agent. There were no significant differences in those who felt a reversal agent would help with adherence among those who did or did not previously take warfarin (28.9% vs 27.6%, respectively, felt it would help a great deal; 20.6 vs 17.7%, respectively, felt it would help a lot).

Patients with adherence scores >80 put more weight on physician recommendations than those with adherence scores <80 (75% vs 53% believed it would help a great deal/a lot; *P* < 0.001), and valued reversal agents more (51% vs 38% believed it would help a great deal/a lot; *P* = 0.03). Forty-three percent of all patients did not inform their physicians about their nonadherence. Though not reaching statistical significance (*P* = 0.06), likeliness of informing physicians of nonadherence appeared inversely proportional to adherence rates (56% of those with adherence scores <60 did not tell physicians, compared to 44% with adherence scores 60-79 and 39% with scores ≥80).

## Discussion

This cross-sectional survey of patients taking apixaban for AF evaluated both data that would be available in EHR systems and patient-reported reasons for nonadherence that are not included in EHR data. Our novel findings are that demographic and health information that would typically be available in EHR systems and administrative databases (such as age, sex, and race/ethnicity) were not associated with patient-reported nonadherence, while forgetfulness was the single most frequently cited reason for nonadherence. Strategies to address forgetfulness, such as dose administration aids, alarms, and reminder messages are available and have been reviewed in other studies.^33^, ^34^, ^35^ While patients with mild nonadherence (adherence scores≥80) mostly indicated that their nonadherence was unintentional (due to forgetfulness), those with poor adherence (adherence scores <80) more frequently cited intentional nonadherence related to their attitudes and beliefs. We also found that patients with the lowest adherence were least likely to disclose this information to their prescriber. Although the lack of monitoring of DOAC anticoagulation has been considered a potential contributor to greater patient satisfaction and adherence, patients suggested that strategies they believed would help a great deal/a lot to enhance their adherence included doing bloodwork to assess medication efficacy.

We also quantified reasons for nonadherence that are general concerns identified with anticoagulation with warfarin as well as DOACs.^17^ Patients in this study who reported the poorest adherence (adherence scores <60) more often feared severe bleeding, with 40% citing fear of severe bleeding as a reason for their nonadherence, and almost one-quarter indicating that it was their primary reason for nonadherence. These patients also more often questioned the need for apixaban when they did not experience symptoms of AF, with about one-third of those with adherence scores <60 citing lack of symptoms as a reason for their nonadherence, and about 10% indicating that it was their primary reason for nonadherence. Patients with adherence scores <60 also more often believed they did not need to take apixaban and noted that they experienced bothersome minor bleeding. Eighty percent of all patients and two-thirds of those with adherence scores <60 in our study worried more about having a stroke than about severe bleeding. Coupled with our findings that patients with adherence scores <60 more often failed to recognize that apixaban prevents strokes in those with AF, this study suggests a potential role for better education regarding the indication for apixaban use.

Though the knowledge gaps we identified have been reported by both providers and patients,^36^^,^^37^ they have not been studied with respect to adherence. Many health care systems do not or cannot track adherence by prescription fills and refills. Self-reported medication nonadherence has been associated with chronic coronary disease outcomes,^38^ underscoring the utility of self-reported measures of adherence. We used a previously validated 3-item measure of adherence^30^ to identify patients for whom addressing adherence related to knowledge gaps may be desirable.

Our findings reinforce the need to identify patients with poor adherence, elicit information about the reasons underlying their nonadherence, and address their individual concerns. Patients with adherence scores <60 cited the most reasons for their nonadherence, indicating that multiple avenues for intervention may exist for these patients. Strategies that patients with adherence scores <80 suggested might be most helpful for them included laboratory monitoring to determine the efficacy of apixaban. These findings are consistent with results from other studies showing that patients want monitoring of DOACs.^39^^,^^40^ DOAC concentration measurements with anti-factor Xa activity assays are available in hospital and national laboratories (Quest, Mayo, LabCorp), but routine measurements of DOAC concentrations are not currently recommended except in certain situations. For example, monitoring may be beneficial in patients with very high or low body mass indexes, or with renal impairment.^41^^,^^42^ Many consider DOACs to be advantageous because regular monitoring is not needed, but for certain patients with nonadherence, monitoring might serve to boost confidence in the effect of DOACs and increase adherence. Patient knowledge about the existence of reversal agents, the frequency of physician communication about these agents, and the influence of knowledge about the agents on adherence requires additional exploration, as does the relationship of these interventions on patient adherence. Qualitative work suggests that patients and providers support educating patients about DOACs,^43^ and indeed, patient education has successfully increased adherence to other medications, including to warfarin.^33^^,^^44^ However, studies focused on DOAC adherence are needed. In addition, all patients surveyed indicated that greater physician emphasis on not missing doses would be a motivator for adherence.

Importantly, over one-half of those with adherence scores <60 failed to inform their physicians about their nonadherence. These findings suggest the need for routine queries about adherence. A prior study showed that physicians infrequently ask patients directly about medication nonadherence,^45^ suggesting a potential role for pharmacists and medical assistants in filling gaps in identification. Once the healthcare team identifies nonadherence, physicians could delve deeper into the reason(s) for nonadherence and tailor additional discussions to patient needs. Coupling discussions with strong physician recommendations about the importance of adherence may influence even those patients who forget to take their medication to be more attentive to adherence.

This study has several limitations. The number of respondents who reported nonadherence to apixaban was small. Patients in this study were primarily White and had at least a college education, which may limit the generalizability of our results. We did not assess patient insurance coverage or household income. Individual EHRs were not reviewed, so we could not assess effects related to providers, specialized clinics or educational programs, duration of AF or apixaban use, or use of other DOACs prior to apixaban. Though we asked patients whether they had sought urgent or emergency care for bleeding in the past 12 months, we did not assess whether they had ever experienced a significant bleed. As this is an observational study, unobserved confounders may exist.

Actual rates of nonadherence to apixaban may differ from what we found in our study. Survey invitations informed patients that the study examined patient decisions about blood thinners, so those who chose not to take or to stop blood thinners may have been less motivated to participate, thus leading to underestimation of nonadherence. Though we used a validated measure of nonadherence, patients self-reported on their nonadherence. In addition, we lacked information on whether nonadherence was primary or secondary. Different trajectories of nonadherence to anticoagulants in patients with AF may result from different underlying reasons. This cross-sectional study does not allow for identification of these trajectories. Lastly, it is difficult to evaluate how the patient perceptions we identified might translate to adherence to medications with different dosing schedules (eg, once daily) or to other medications patients might be taking. Reductions in daily dosing can improve medication adherence.^46^ Yet in this study, in which most participants took multiple medications, those taking more medications had significantly higher adherence than those taking fewer medications, suggesting that twice daily dosing may not influence adherence in older patients with certain medical conditions.

## Conclusions

This study begins to answer questions that move beyond examining whether AF patients are appropriately prescribed anticoagulation for stroke prevention to understanding levers for improving adherence in patients receiving DOAC prescriptions to prevent strokes. Multiple studies have shown that nonadherence is prevalent in patients prescribed DOACs yet studies based on analyses of administrative data have failed to determine the underlying reasons. The current work indicates that almost half of patients with nonadherence do not disclose their nonadherence to their healthcare providers, and those with the poorest adherence question the need for anticoagulation, especially if they have no symptoms. Fear of bleeding and the cost of medications can contribute to nonadherence. Prescribers as well as patients have a role in decreasing nonadherence. Prescribers must assess nonadherence and address potential patient misperceptions about the goals of therapy with anticoagulation in AF, help patients address difficulties with cost, and promote the importance of adherence to DOACs for stroke prevention.

PERSPECTIVES **COMPETENCY IN MEDICAL KNOWLEDGE 1:** In addition to forgetfulness, patient-reported contributors to nonadherence include beliefs that the medication is not needed, medication cost, and fear of severe bleeding.

**COMPETENCY IN MEDICAL KNOWLEDGE 2:** Patients with poor adherence to anticoagulation with apixaban often do not tell their providers about their nonadherence.

**COMPETENCY IN INTERPERSONAL AND COMMUNICATION SKILLS:** It is important to communicate that the goal of anticoagulation in AF is stroke prevention and not symptom control, initiate discussions regarding adherence, assess nonadherence, and address reasons underlying patient nonadherence if present.

**TRANSLATIONAL OUTLOOK 1:** Assessing adherence can facilitate focused efforts on likely underlying contributors. Information is needed on the real-life effectiveness of patient-identified strategies to promote adherence to apixaban and other DOACs.

**TRANSLATIONAL OUTLOOK 2:** Studies should develop and examine interventions to efficiently assess and counsel patients about adherence in a patient-centered manner.

## Funding support and author disclosures

This project was funded by the 10.13039/100002491Bristol-Myers Squibb and 10.13039/100004319Pfizer, as part of the American Thrombosis Investigator Initiated Research Program (ARISTA-USA). It was also supported in part by the 10.13039/100006108National Center for Advancing Translational Sciences, 10.13039/100000002National Institutes of Health, through UCLA 10.13039/100006975CTSI UL1TR001881 and UCSF 10.13039/100006975CTSI UL1TR001872. Its contents are solely the responsibility of the authors and do not necessarily represent the official views of the NIH.

Drs Tarn and Schwartz have been funded by the BMS/Pfizer Alliance ARISTA-USA to conduct unrelated research studies. Dr Schwartz has participated in Cardiovascular Advisory Councils to the BMS/Pfizer Medical Alliance. All other authors have reported that they have no relationships relevant to the contents of this paper to disclose.

## References

[1] Fuster V., Rydén L.E., Cannom D.S. (2006). ACC/AHA/ESC 2006 guidelines for the management of patients with atrial fibrillation: a report of the American College of Cardiology/American Heart Association Task Force on practice guidelines and the European Society of Cardiology Committee for practice guidelines (Writing Committee to Revise the 2001 guidelines for the management of patients with atrial fibrillation): developed in collaboration with the European Heart Rhythm Association and the Heart Rhythm Society. J Am Coll Cardiol.

[2] Ogilvie I.M., Newton N., Welner S.A., Cowell W., Lip G.Y. (2010). Underuse of oral anticoagulants in atrial fibrillation: a systematic review. Am J Med.

[3] Mohan A., Wanat M.A., Abughosh S.M. (2019). Medication taking behaviors in patients taking warfarin versus direct oral anticoagulants: a systematic review. Expert Rev Cardiovasc Ther.

[4] Kirchhof P., Benussi S., Kotecha D. (2016). 2016 ESC guidelines for the management of atrial fibrillation developed in collaboration with EACTS. Kardiol Pol.

[5] Colacci M., Tseng E.K., Sacks C.A., Fralick M. (2020). Oral anticoagulant utilization in the United States and United Kingdom. J Gen Intern Med.

[6] Camm A.J., Accetta G., Ambrosio G. (2017). Evolving antithrombotic treatment patterns for patients with newly diagnosed atrial fibrillation. Heart.

[7] Steinberg B.A., Gao H., Shrader P. (2017). International trends in clinical characteristics and oral anticoagulation treatment for patients with atrial fibrillation: results from the GARFIELD-AF, ORBIT-AF I, and ORBIT-AF II registries. Am Heart J.

[8] January C.T., Wann L.S., Alpert J.S. (2014). 2014 AHA/ACC/HRS guideline for the management of patients with atrial fibrillation: a report of the American College of Cardiology/American Heart Association Task Force on practice guidelines and the Heart Rhythm Society. J Am Coll Cardiol.

[9] Salmasi S., Loewen P.S., Tandun R., Andrade J.G., Vera M.A.D. (2020). Adherence to oral anticoagulants among patients with atrial fibrillation: a systematic review and meta-analysis of observational studies. BMJ Open.

[10] Deitelzweig S., Di Fusco M., Kang A. (2021). Real-world persistence to direct oral anticoagulants in patients with atrial fibrillation: a systematic review and network meta-analysis. Curr Med Res Opin.

[11] Banerjee A., Benedetto V., Gichuru P. (2020). Adherence and persistence to direct oral anticoagulants in atrial fibrillation: a population-based study. Heart.

[12] Ferroni E., Gennaro N., Costa G. (2019). Real-world persistence with direct oral anticoagulants (DOACs) in naïve patients with non-valvular atrial fibrillation. Int J Cardiol.

[13] Smits E., Andreotti F., Houben E. (2022). Adherence and persistence with once-daily vs twice-daily direct oral anticoagulants among patients with atrial fibrillation: real-world analyses from the Netherlands, Italy and Germany. Drugs Real World Outcomes.

[14] Hernandez I., He M., Chen N., Brooks M.M., Saba S., Gellad W.F. (2019). Trajectories of oral anticoagulation adherence among medicare beneficiaries newly diagnosed with atrial fibrillation. J Am Heart Assoc.

[15] Miyazaki M., Nakashima A., Nakamura Y. (2018). Association between medication adherence and illness perceptions in atrial fibrillation patients treated with direct oral anticoagulants: an observational cross-sectional pilot study. PLoS One.

[16] Osasu Y.M., Cooper R., Mitchell C. (2021). Patients’ and clinicians’ perceptions of oral anticoagulants in atrial fibrillation: a systematic narrative review and meta-analysis. BMC Fam Pract.

[17] Pandya E.Y., Bajorek B. (2017). Factors affecting patients' perception on, and adherence to, anticoagulant therapy: anticipating the role of direct oral anticoagulants. Patient.

[18] Toorop M.M.A., van Rein N., Nierman M.C. (2020). Self-reported therapy adherence and predictors for nonadherence in patients who switched from vitamin K antagonists to direct oral anticoagulants. Res Pract Thromb Haemost.

[19] Skanes A.C., Gula L.J. (2021). Can we anticipate nonadherence to anticoagulation?: elegant modeling, but no clear predictions. J Am Coll Cardiol.

[20] Salmasi S., De Vera M.A., Safari A. (2021). Longitudinal oral anticoagulant adherence trajectories in patients with atrial fibrillation. J Am Coll Cardiol.

[21] Salmasi S., Safari A., Kapanen A. (2022). Oral anticoagulant adherence and switching in patients with atrial fibrillation: a prospective observational study. Res Social Adm Pharm.

[22] Gumbinger C., Holstein T., Stock C., Rizos T., Horstmann S., Veltkamp R. (2015). Reasons underlying non-adherence to and discontinuation of anticoagulation in secondary stroke prevention among patients with atrial fibrillation. Eur Neurol.

[23] Tarn D.M., Shih K.J., Schwartz J.B. (2021). Reasons for nonadherence to the direct oral anticoagulant apixaban for atrial fibrillation. J Am Geriatr Soc.

[24] Khera R., Valero-Elizondo J., Das S.R. (2019). Cost-related medication nonadherence in adults with atherosclerotic cardiovascular disease in the United States, 2013 to 2017. Circulation.

[25] Van Alsten S.C., Harris J.K. (2020). Cost-related nonadherence and mortality in patients with chronic disease: a multiyear investigation, national health interview survey, 2000-2014. Prev Chronic Dis.

[26] Kaufman B.G., Kim S., Pieper K. (2018). Disease understanding in patients newly diagnosed with atrial fibrillation. Heart.

[27] McCabe P.J., Schad S., Hampton A., Holland D.E. (2008). Knowledge and self-management behaviors of patients with recently detected atrial fibrillation. Heart Lung.

[28] Chew L.D., Bradley K.A., Boyko E.J. (2004). Brief questions to identify patients with inadequate health literacy. Fam Med.

[29] Chew L.D., Griffin J.M., Partin M.R. (2008). Validation of screening questions for limited health literacy in a large VA outpatient population. J Gen Intern Med.

[30] Wilson I.B., Lee Y., Michaud J., Fowler F.J., Rogers W.H. (2016). Validation of a new three-item self-report measure for medication adherence. AIDS Behav.

[31] Wilson I.B., Fowler F.J., Cosenza C.A. (2014). Cognitive and field testing of a new set of medication adherence self-report items for HIV care. AIDS Behav.

[32] Nguyen T.-M.-U., Caze A.L., Cottrell N. (2014). What are validated self-report adherence scales really measuring?: a systematic review. Br J Clin Pharmacol.

[33] Cross A.J., Elliott R.A., Petrie K., Kuruvilla L., George J. (2020). Interventions for improving medication-taking ability and adherence in older adults prescribed multiple medications. Cochrane Database Syst Rev.

[34] George J., Elliott R.A., Stewart D.C. (2008). A systematic review of interventions to improve medication taking in elderly patients prescribed multiple medications. Drugs Aging.

[35] Maki K., Harris K. (2021). Communication Strategies to Improve Medication Adherence: A Systematic Review of Literature. RCR [Internet].

[36] Salmasi S., De Vera M.A., Barry A. (2019). Assessment of condition and medication knowledge gaps among atrial fibrillation patients: a systematic review and meta-analysis. Ann Pharmacother.

[37] Salmasi S., Kwan L., MacGillivray J. (2019). Assessment of atrial fibrillation patients' education needs from patient and clinician perspectives: a qualitative descriptive study. Thromb Res.

[38] Garcia R.A., Spertus John A., Benton Mary C. (2022). Association of medication adherence with health outcomes in the ISCHEMIA trial. J Am Coll Cardiol.

[39] Mull H.J., Shin M.H., Engle R.L. (2020). Veterans perceptions of satisfaction and convenience with anticoagulants for atrial fibrillation: warfarin versus direct oral anticoagulants. Patient Prefer Adherence.

[40] Bajorek B., Saxton B., Anderson E., Chow C.K. (2018). Patients' preferences for new versus old anticoagulants: a mixed-method vignette-based study. Eur J Cardiovasc Nurs.

[41] McRae H.L., Militello L., Refaai M.A. (2021). Updates in anticoagulation therapy monitoring. Biomedicines.

[42] Zhang H., Liu Z., Mu G. (2020). Diagnostic performance of coagulation indices for direct oral anticoagulant concentration. Thromb Res.

[43] Wang M., Swinton M., Troyan S. (2022). Perceptions of patients and healthcare providers on patient education to improve oral anticoagulant management. J Eval Clin Pract.

[44] Clarkesmith D.E., Pattison H.M., Lip G.Y., Lane D.A. (2013). Educational intervention improves anticoagulation control in atrial fibrillation patients: the TREAT randomised trial. PLoS One.

[45] Tarn D.M., Mattimore T.J., Bell D.S., Kravitz R.L., Wenger N.S. (2012). Provider views about responsibility for medication adherence and content of physician-older patient discussions. J Am Geriatr Soc.

[46] Ingersoll K.S., Cohen J. (2008). The impact of medication regimen factors on adherence to chronic treatment: a review of literature. J Behav Med.

