# Supplementary material for: Reasons for Nonadherence to the Direct Oral Anticoagulant Apixaban: A Cross-Sectional Survey of Atrial Fibrillation Patients
Source: JACC Adv. 2023 Jan 27;2(1):100175. doi: 10.1016/j.jacadv.2022.100175 (PMC11198726; doi:10.1016/j.jacadv.2022.100175)
Supplement: Appendix 1 [file mmc1.pdf]

Appendix 1. Survey Instrument (Electronic Version)

Eligibility Screening

Doctors at UCLA and UCSF need your help to improve patient care! You will receive a $25 electronic gift card if you are eligible and complete this survey about atrial fibrillation treatment.

Our goal is to hear your opinions. This study is run by doctors at UCLA and UCSF medical schools.

Your answers are confidential. They will not be shared with anyone outside the research team. Your doctor will not know if you participated. The survey will take about 10-15 minutes. The study is being funded by Bristol Myers Squibb.

For each question, please read all the answer choices carefully. Select **only one** answer for each question, unless otherwise noted.

We really appreciate your help!

Derjung Mimi Tarn, MD, PhD
Department of Family Medicine
David Geffen School of Medicine at UCLA

Janice B. Schwartz, MD
Department of Bioengineering & Therapeutic Sciences
University of California, San Francisco

Please press the arrow button in the bottom right-hand corner to see if you're eligible.

Do you have atrial fibrillation or atrial flutter? (Select one)

- ◯ No

- Yes
- I am not sure

Did your doctor tell you if your atrial fibrillation or atrial flutter is:
(Select one)

- Paroxysmal (comes and goes)
- Chronic, permanent, or persistent (all the time)
- I do not know or I am not sure

Have you ever been prescribed Eliquis (apixaban)? (Select one)

- No
- Yes

Which of the following best describes how you take Eliquis (apixaban)? (Select one)

- I never miss any doses
- I sometimes miss a dose
- I often miss a dose
- I am not taking Eliquis

If you are not taking Eliquis (apixaban) or sometimes miss or skip a dose, was this due to a doctor's advice? (Select one)

- No
- Yes

In the last 30 days, on how many days did you miss at least one dose of any of your Eliquis (apixaban)? Please choose the number of days.

In the last 30 days, **how often** did you take your Eliquis (apixaban) in the way you were supposed to? (Select one)

- Never
- Rarely
- Sometimes
- Usually
- Almost always
- Always

In the last 30 days, **how good** a job did you do at taking your Eliquis (apixaban) in the way you were supposed to? (Select one)

- Very poor
- Poor
- Fair
- Good
- Very good
- Excellent

Survey (for eligible respondents only)

Congratulations! You are eligible for the survey! Please follow the directions below carefully.

What is your age? (Please select number in years)

How do you identify? (Select one)

- ○ Male
- ○ Female
- ○ Other

Are you of Hispanic, Latino, or Spanish origin? (Select one)

- ○ Yes
- ○ No

Which of the following best describes you? (Select all that apply)

- ☐ American Indian or Alaska Native
- ☐ Asian
- ☐ Black or African American
- ☐ Native Hawaiian or other Pacific Islander
- ☐ White or Caucasian
- ☐ ________ Other (Write in):

What is the highest degree or level of school you have completed? (Select one)

- ○ High school diploma or less
- ○ Some college or associate degree
- ○ Bachelor's degree
- ○ Advanced degree (for example, master's, doctoral degree, or professional degree)

Have you ever taken Coumadin (warfarin)? (Select one)

- ◯ Yes
- ◯ No
- ◯ I am not sure

Indicate if you had or have any of the below. (Mark “Yes”, “No”, or "Not sure" for each)

	Yes	No	Not sure
Heart failure	◯	◯	◯
High blood pressure	◯	◯	◯
Diabetes	◯	◯	◯
Stroke or TIA (transient ischemic attack)	◯	◯	◯
Blood clots	◯	◯	◯
Heart attack	◯	◯	◯
Peripheral artery disease	◯	◯	◯
Calcium or plaque in the aorta on a CT scan	◯	◯	◯

Below are some reasons a person may not take Eliquis (apixaban) exactly the way their doctor advised. Please mark **all of the reasons** you do not take Eliquis (apixaban) exactly the way your doctor advised. (Select **ALL** that apply)

- ☐ I forget to take Eliquis
- ☐ Eliquis costs too much
- ☐ I am scared that Eliquis might cause bleeding that is severe or that I cannot control
- ☐ I have had minor bleeding from Eliquis that bothers me
- ☐ I have had bruising from Eliquis that bothers me
- ☐ I do not always have symptoms of atrial fibrillation
- ☐ It is okay to miss taking Eliquis here or there because it stays in my body

- ☐ I worry that Eliquis will cause problems with my other medicine
- ☐ I am not supposed to drink alcohol when I take Eliquis
- ☐ I do not think I need to take Eliquis
- ☐ ________ Other (write in):

Please select **the MAIN reason** you do not take Eliquis exactly the way your doctor advised. (Select one)

- ○ » I forget to take Eliquis
- ○ » Eliquis costs too much
- ○ » I am scared that Eliquis might cause bleeding that is severe or that I cannot control
- ○ » I have had minor bleeding from Eliquis that bothers me
- ○ » I have had bruising from Eliquis that bothers me
- ○ » I do not always have symptoms of atrial fibrillation
- ○ » It is okay to miss taking Eliquis here or there because it stays in my body
- ○ » I worry that Eliquis will cause problems with my other medicine
- ○ » I am not supposed to drink alcohol when I take Eliquis
- ○ » I do not think I need to take Eliquis
- ○ » Other (write in):

Did you tell your doctor that you do not take Eliquis exactly the way they advised? (Select one)

- ○ Yes
- ○ No

Please indicate how worried or not worried you are about each item below: (Mark one answer for each)

	Extremely worried	Moderately worried	Somewhat worried	Slightly worried	Not at all worried

	Extremely worried	Moderately worried	Somewhat worried	Slightly worried	Not at all worried
Eliquis might cause bleeding that is severe or that you cannot control	○	○	○	○	○
Eliquis will cause problems with your other medicine	○	○	○	○	○
Not being able to drink alcohol when you take Eliquis	○	○	○	○	○

How much do you agree or disagree with each item below?
(Mark one answer for each)

	Strongly agree	Somewhat agree	Neither agree nor disagree	Somewhat disagree	Strongly disagree
Eliquis prevents strokes in people with atrial fibrillation	○	○	○	○	○
Eliquis helps control the symptoms of atrial fibrillation	○	○	○	○	○
People can be in atrial fibrillation without feeling it	○	○	○	○	○
It is okay to miss taking Eliquis here or there because it stays in my body	○	○	○	○	○
I do not think I need to take Eliquis	○	○	○	○	○
People's own behaviors can make their atrial fibrillation worse	○	○	○	○	○
What people eat or drink can make atrial fibrillation worse	○	○	○	○	○

During the past 12 months, have you skipped Eliquis doses to save money? (Select one)

- Yes
- No

During the past 12 months, have you taken less Eliquis to save money? (Select one)

- Yes
- No

During the past 12 months, have you delayed filling Eliquis to save money? (Select one)

- Yes
- No

During the past 12 months, have you asked your doctor for a lower-cost medicine (in place of Eliquis) to save money? (Select one)

- Yes
- No

The questions below ask about any bleeding you may have had in the past 12 months.

In the past 12 months, did you go to an emergency room or seek urgent care for bleeding? (Select one)

- Yes
- No

In the past 12 months, how often have you had a nosebleed that was hard to stop? (Select one)

- Often
- Sometimes
- Seldom
- Never

How much do your nosebleeds bother you? (Select one)

- A great deal
- A lot
- A moderate amount
- A little
- None at all

In the past 12 months, how often have you had bleeding from a cut that was hard to stop? (Select one)

- Often
- Sometimes
- Seldom
- Never

How much does bleeding from cuts that are hard to stop bother you? (Select one)

- A great deal
- A lot
- A moderate amount
- A little
- None at all

In the past 12 months, how often have you had bruising? (Select one)

- Often
- Sometimes
- Seldom
- Never

How much does bruising bother you? (Select one)

- A great deal
- A lot
- A moderate amount
- A little
- None at all

In the past 12 months, did you talk to a doctor about your bleeding or bruising? (Select one)

- Yes
- No

Think about your risk of having a stroke in the next year. How **high** does your risk need to be for you to take Eliquis? (Select one)

- More than 1 in 2 chance (50%)
- More than 1 in 4 chance (25%)
- More than 1 in 10 chance (10%)
- More than 1 in 20 chance (5%)
- More than 1 in 50 chance (2%)
- My risk of stroke would not change my Eliquis use

- I don’t know

Which of the following scares you more? (Select one)

- Having a stroke
- Having bleeding that is severe or that I cannot control
- I don't know

How much would each of the following help you to take Eliquis exactly the way your doctor advised:
(Mark one answer for each)

	A great deal	A lot	A moderate amount	A little	None at all
Your doctor told you it was very important to never miss taking it	○	○	○	○	○
You got Eliquis for free	○	○	○	○	○
Serious or life-threatening bleeding from Eliquis could be reversed by a medicine (reversal agent) given after bleeding occurs	○	○	○	○	○
You could do a blood test to see how well Eliquis is working	○	○	○	○	○

How much would reading your medical records help you to take Eliquis exactly the way your doctor advised? (Select one)

- A great deal
- A lot
- A moderate amount

- A little
- None at all

A prescription medicine is one that you CANNOT buy on your own. A doctor needs to prescribe (or give) it to you.

How many prescription medicines do you take regularly by mouth or as a shot? Give your best estimate. Please select 0 if you do not take any prescription medicines.

In the past 12 months, have you taken any of the following? (Mark one answer for each)

	Yes	No	Not sure
Aspirin	○	○	○
Ibuprofen, Advil, Motrin, naproxen, Naprosyn, Aleve, or other non-steroidal anti-inflammatory medicine	○	○	○
Tylenol (acetaminophen)	○	○	○
Fish oil	○	○	○
Turmeric	○	○	○
Plavix (clopidogrel), Effient (prasugrel), Brilinta (ticagrelor)	○	○	○
Esomeprazole (Nexium), lansoprazole (Prevacid), omeprazole (Prilosec), pantoprazole (Protonix), or rabeprazole (AcipHex)	○	○	○

How confident are you filling out medical forms by yourself? (Select one)

- Extremely
- Quite a bit
- Somewhat
- A little bit
- Not at all

During the past 12 months have you had **significant problems** with **your memory or thinking** that interferes with your ability to do things that you regularly do, such as taking care of your home, managing your finances, or keeping up with TV programs?

- Yes
- No

Did somebody help you fill out this survey? (Select one)

- Yes
- No

Thank you for your time. May we contact you for future studies?

- Yes
- No

Please select from the options below. If a gift card is selected, you can expect to receive it in 6-8 weeks. (Select one)

- $25 Amazon electronic gift card
- $25 Target electronic gift card

- ○ No gift card. I wish to donate my time to the study.

Powered by Qualtrics
